# Weak Association Between the Glutamate Decarboxylase 1 Gene (GAD1) and Schizophrenia in Han Chinese Population

**DOI:** 10.3389/fnins.2021.677153

**Published:** 2021-06-21

**Authors:** Luwen Zhang, Zhen Li, Qing Liu, Minglong Shao, Fuping Sun, Xi Su, Meng Song, Yan Zhang, Minli Ding, Yanli Lu, Jiewei Liu, Yongfeng Yang, Ming Li, Wenqiang Li, Luxian Lv

**Affiliations:** ^1^Henan Mental Hospital, The Second Affiliated Hospital of Xinxiang Medical University, Xinxiang, China; ^2^Henan Key Lab of Biological Psychiatry, Xinxiang Medical University, Xinxiang, China; ^3^International Joint Research Laboratory for Psychiatry and Neuroscience of Henan, Xinxiang, China; ^4^Key Laboratory of Animal Models and Human Disease Mechanisms of the Chinese Academy of Sciences and Yunnan Province, Kunming Institute of Zoology, Chinese Academy of Sciences, Kunming, China; ^5^Henan Province People’s Hospital, Zhengzhou, China

**Keywords:** GAD1, schizophrenia, single-nucleotide polymorphisms, Han Chinese, genetic association

## Abstract

**Objectives:**

Schizophrenia (SZ) is a complex psychiatric disorder with high heritability, and genetic components are thought to be pivotal risk factors for this illness. The glutamate decarboxylase 1 gene (GAD1) was hypothesized to be a candidate risk locus for SZ given its crucial role in the GABAergic neurotransmission system, and previous studies have examined the associations of single nucleotide polymorphisms (SNPs) spanning the GAD1 gene with SZ. However, inconsistent results were obtained. We hence examined the associations between GAD1 SNPs and SZ in two independent case-control samples of Han Chinese ancestry.

**Materials and Methods:**

Two Han Chinese SZ case-control samples, referred as the discovery sample and the replication sample, respectively, were recruited for the current study. The discovery sample comprised of 528 paranoid SZ cases (with age of first onset ≥ 18) and 528 healthy controls; the independent replication sample contained 1,256 early onset SZ cases (with age of first onset < 18) and 2,661 healthy controls. Logistic regression analysis was performed to examine the associations between GAD1 SNPs and SZ.

**Results:**

Ten SNPs covering GAD1 gene were analyzed in the discovery sample, and two SNPs showed nominal associations with SZ (rs2241165, *P* = 0.0181, OR = 1.261; rs2241164, *P* = 0.0225, OR = 1.219). SNP rs2241164 was also nominally significant in the independent replication sample (*P* = 0.0462, OR = 1.110), and the significance became stronger in a subsequent meta-analysis combining both discovery and replication samples (*P* = 0.00398, OR = 1.138). Nevertheless, such association could not survive multiple corrections, although the effect size of rs2241164 was comparable with other SZ risk loci identified in genome-wide association studies (GWAS) in Han Chinese population. We also examined the associations between GAD1 SNPs and SZ in published datasets of SZ GWAS in East Asians and Europeans, and no significant associations were observed.

**Conclusion:**

We observed weak associations between GAD1 SNPs and risk of SZ in Han Chinese populations. Further analyses in larger Han Chinese samples with more detailed phenotyping are necessary to elucidate the genetic correlation between GAD1 SNPs and SZ.

## Introduction

Schizophrenia (SZ) is a severe mental illness that affects approximately 1% of human populations and imposes a high burden on family and society (Huang et al., 2019; McCutcheon et al., 2020). This illness is characterized by positive symptoms (auditory hallucinations, delusions, and disorganized speech), negative symptoms (blunted affect and social withdrawal), and cognitive dysfunctions (Marder and Cannon, 2019). Although the etiology of SZ remains largely unclear, it is believed to be resulted from a combinatorial impact of complex genetic and environmental factors (Gottesman and Shields, 1967). Studies have also found that genetic factor play important roles in the onset and development of SZ (Sullivan et al., 2003; Lichtenstein et al., 2009).

Recently, the Schizophrenia Working Group of the Psychiatric Genomics Consortium (PGC) has performed the SZ genome-wide association studies (GWAS) with continuously accumulating samples of European ancestry (Schizophrenia Working Group of the Psychiatric Genomics Consortium,, 2014; Pardinas et al., 2018), and identified substantial single nucleotide polymorphisms (SNPs) significantly associated with risk of SZ. In the genetically divergent Han Chinese or East Asian populations, GWAS have also reported multiple genome-wide significant loci associated with SZ (Li et al., 2017; Yu et al., 2017; Lam et al., 2019). Overall, these GWAS have provided essential information regarding SZ pathogenesis, and have confirmed some classical pathological hypotheses of the illness, such as the dopamine hypothesis and neurodevelopmental hypothesis.

Glutamic acid decarboxylase (GAD) catalyzes synthesis of the inhibitory neurotransmitter gamma-amino butyric acid (GABA) in the brain (Akbarian and Huang, 2006). GABA is essentially implicated in the pathophysiology of SZ, particularly in three core symptoms: learning, memory, and executive functions (Yoon et al., 2010; Schmidt and Mirnics, 2015). Molecules related to the GABA signaling transduction pathway are widely interacting with other neurotransmitters and signaling pathways implicated in the development of SZ (Represa and Ben-Ari, 2005; Fazzari et al., 2010). To the mammalian, GABA is synthesized by two isoforms of glutamic acid decarboxylase, and GAD1 is the rate-limiting enzyme (Erlander et al., 1991; Szabo et al., 1994). Some autopsy studies have found that both mRNA and protein levels of GAD1 are lower in multiple cortical areas and hippocampus of SZ patients (Akbarian et al., 1995; Fatemi et al., 2005; Hashimoto et al., 2008; Curley et al., 2011; Hyde et al., 2011; Joshi et al., 2012; Ruzicka et al., 2015; Atagun et al., 2017; Tao et al., 2018; Kilonzo et al., 2020). For example, dysfunction of GABAergic neurotransmission has been observed in SZ through investigations of human postmortem brain tissues and *in vivo* levels of GABA (de Jonge et al., 2017); alternative splicing of the GAD1 gene also likely contributes to GABA dysfunction in the brains of schizophrenics (Zhang et al., 2021). Therefore, GAD1 is likely involved in the pathogenesis of SZ. To date, several previous studies have explored the genetic associations between GAD1 and SZ in humans (Addington et al., 2005; Straub et al., 2007). A study in European populations revealed that the GAD1 gene was associated with childhood-onset SZ and reduction in the volume of gray matter cortex of the cerebrum (Addington et al., 2005). However, few studies have examined the link between GAD1 and SZ in Han Chinese populations.

In this study, using two independent case-control samples recruited from Han Chinese populations, we aimed to systematically investigate the associations of GAD1 SNPs with SZ, and hope to provide evidence for the involvement of this gene in the heritable risk of SZ in Han Chinese subjects.

## Materials and Methods

### Ethnic Statement and Study Approval

The protocol was approved by the Ethical Committee of the Second Affiliated Hospital of Xinxiang Medical University. Written informed consents were obtained from all participants after the objectives and procedures of the study were fully explained.

### Description About Discovery Sample

In the discovery sample, 900 SZ patients were recruited from inpatients of the Second Affiliated Hospital of Xinxiang Medical University, whereas and 900 healthy controls were recruited from employees of the hospital, visitors of the physical examination center and college students of the Xinxiang Medical University. All participants were of Han Chinese origin from northern Henan Province. Diagnoses were made according to the consensus of at least two experienced psychiatrists following the diagnostic criteria for SZ in the Diagnostic and Statistical Manual of Mental Disorders-Fourth Edition IV (DSM-IV) (1994). To ensure inter-rater consistency, all participating psychiatrists were trained every 6 months in which diagnoses and test results were compared using videotaped demonstration interviews. Individuals with complex medical complications history, organic brain diseases, other major psychiatric disorders, or substance dependence were excluded. To attain the aim of achieving a gender-equalized number of patients with paranoid SZ, all female patients with paranoid SZ who did not fulfill the exclusion criteria was included as well as an equal number of male patients with the same diagnosis. The age of first manifestation of positive symptoms was defined as the age at onset of SZ (White et al., 2006). Age at onset was derived from the Comprehensive Assessment of Symptoms and History (CASH) (Andreasen et al., 1992), and all cases had onset of SZ in adulthood (defined as age of first onset ≥ 18). The controls were evaluated by psychiatrists using a simple unstructured interview to exclude those with a personal or family history of mental or neurological diseases. Cases (528 cases) and controls (528 cases) included for the current study were well matched in age, gender (264 males and 264 females pre group, mean age 27.73 ± 8.01 years old, respectively), and ethnicity.

GAD1 SNPs were selected for the present analyses based on the following criteria: (1) all SNPs covering the genomic region of GAD1 gene were subjected to functional analysis using the FASTSNP online service (Yuan et al., 2006), and only SNPs with highly ranked risk and a minor allele frequency (MAF) ≥ 0.05 in the Chinese Beijing population according to the HapMap database were selected (International HapMap Consortium, 2003); (2) tag SNPs were chosen based on the aggressive tagging algorithm (*r*^2^ ≥ 0.80, MAF ≥ 0.05) using genotype data from the HapMap dataset as implemented in Haploview v4.1 (Carlson et al., 2004; Barrett et al., 2005); (3) SNPs selected with the above two criteria and had Illumina design scores over 0.6 were proceeded for further analyses.

Peripheral venous blood was drawn from each participant and collected with vacutainer tubes containing anticoagulant ethylene diamine tetracetic acid (EDTA). Genomic DNA was extracted using Blood Genome DNA Extraction Kit (QIAGEN, Hiden, Germany). SNP genotyping was performed using the Illumina Golden Gate assay on a Bead Station 500G Genotyping System. Genomic DNA samples from cases and controls (250 ng/subject) were randomly sorted and genotyped following the Illumina protocol. All assays were performed blind to diagnosis or genotype. Genotype calls were made using the Genotyping module of the Bead Studio 2.0 software (Illumina, Inc., San Diego, United States). Data was examined for cluster separation using quality scores generated by the Illumina software. SNPs with a Gen Train score b0.4 or a cluster separation score b0.6 were considered poorly genotyped and excluded. For quality control analysis, 96 individuals were also genotyped by Sanger DNA sequencing to ensure the quality of Illumina Golden Gate assay. SNPs significantly deviated from Hardy-Weinberg equilibrium (*P* < 0.05) in the control samples were excluded from further analysis.

### Description About Replication Sample

Summary statistics of the SNPs spanning GAD1 from a recent GWAS of early onset SZ (defined as age of first onset < 18) in 1,256 SZ cases and 2,661 healthy controls of Han Chinese origin (Guo et al., 2021) were retrieved for the replication analysis. SZ cases were assessed with Structured Clinical Interview for DSM-IV Axis I Disorders (SCID), and diagnosis was made based on the Diagnostic and Statistical Manual of Mental Disorders IV (DSM-IV) criteria by at least two experienced psychiatrists. The age at first manifestation of positive symptoms was defined as the age at onset of SZ (White et al., 2006). Age at onset was derived from the Comprehensive Assessment of Symptoms and History (CASH) (Andreasen et al., 1992), and all cases were early onset (defined as age of first onset < 18). SZ cases with neurological diseases (including multiple sclerosis and epilepsy), pervasive developmental disorder (including mental retardation), and other psychiatric disorders (including autism, bipolar disorder, depression, and attention deficit hyperactivity disorder), or with a history of drug abuse and head injury were excluded. The mean onset age of the SZ cases was 14.57 ± 2.27 years old, and 41% cases were males. All controls (55% were males) were healthy volunteers at a mean age of 28.60 ± 7.01 years without family history of any psychiatric disorders. There were no overlapped individuals between the discovery and replication samples, and detailed descriptions about the replication samples can be found in the original study (Guo et al., 2021).

The replication sample subjects were genotyped using Illumina Genome-Wide Asian Screening Array (ASA) Chip. Quality control (QC) analyses were conducted following a previously published pipeline (Anderson et al., 2010). After QC, autosomal biallelic SNPs were proceeded for genotype imputation using minimac3 software (Das et al., 2016) with the 1000 Genomes Project Phase 3 data as the reference panel (Genomes Project Consortium Auton et al., 2015). Eventually, autosomal biallelic SNPs with imputation quality score INFO > 0.8, MAF > 0.01, call rate > 95%, and Hardy-Weinberg equilibrium (HWE) *P* > 1.00 × 10^–5^ in controls and *p* > 1.00 × 10^–10^ in cases were remained. A total of 182 SNPs spanning the coding region of GAD1 gene as well as the ± 20 kb flanking regions were chosen for the present study.

### Statistical Analysis

Hardy-Weinberg equilibrium (HWE), linkage disequilibrium (LD) coefficient (r^2^) and haplotype block were assessed using Haploview v4.1 (Barrett et al., 2005). Logistic regression analysis by adjusting for the significant PCs and sex in the discovery and replication samples was conducted using PLINK (version 1.9) (Purcell et al., 2007) to examine associations of 182 GAD1 SNPs with SZ. SNPs with a *P*-value of 2.75 × 10^–4^ (≈0.05/182) was considered statistically significant. For the meta-analysis combining discovery and replication samples, we used odds ratio (OR) and standard error (SE) of each sample to calculate the pooled OR and the overall 95% confidence intervals (CIs) using an inverse-variance-weighted method under either the fixed-effects model or random-effects model. The Higgins and Thompson *I*^2^ index was used for assessing heterogeneity across samples (Higgins et al., 2003). SNPs showing pronounced heterogeneity (*I^2^*> 75%) underwent meta-analysis in a random-effects model considering the possible test-statistic inflation; SNPs with negligible heterogeneity (*I^2^*≤ 75%) underwent analysis in a fixed-effects model (Winkler et al., 2014). Power analysis was performed using the Power and Sample Size Program software, and the observed OR (1.30) of the most significant SNP in a previous Han Chinese SZ GWAS (i.e., according to SNP rs115070292, *P* = 4.96 × 10^–10^, OR = 1.30) (Yu et al., 2017) was applied in the power analysis (Dupont and Plummer, 1990). Regional plots were made using LocusZoom^1^ (Pruim et al., 2010).

## Results

We herein performed a two-stage analysis, firstly in the discovery sample and then in an independent replication sample, to explore the association of GAD1 gene with SZ in Han Chinese subjects. The discovery sample included 528 SZ patients and 528 controls of Han Chinese ancestry, and ten common SNPs covering GAD1 were genotyped with a genotyping success rate of 99.82%. We have previously examined the population structure of our discovery sample (Li et al., 2012, 2013) and observed no evidence of population stratification. The replication sample comprised of 1,256 SZ cases and 2,661 healthy controls from Han Chinese populations with negligible population stratification (Guo et al., 2021). A total of 182 SNPs spanning the GAD1 gene locus were included.

We conducted a power analysis of the total sample using the following assumptions:1,784 SZ cases and 3,189 controls, as well as two-tailed *P* = 2.75 × 10^–4^. Observed OR of the most significant SNP (i.e., rs115070292, OR = 1.30) from a recent Han Chinese SZ GWAS (Yu et al., 2017) was applied as the input to calculate the statistical power. The present sample size revealed a 78.3% power of detecting a significant allelic association when the allele frequency in general populations reached 0.50.

**FIGURE 1:**
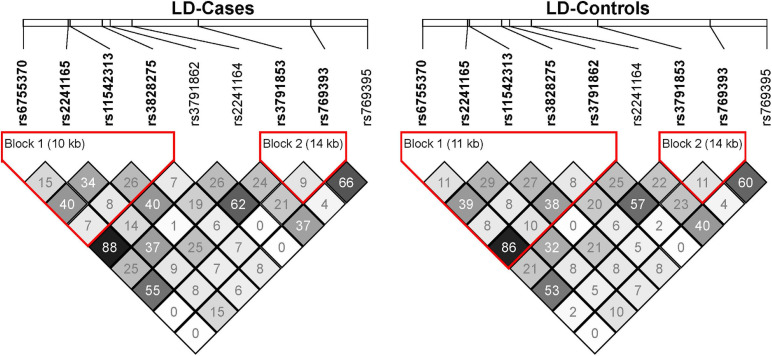
The linkage disequilibrium (LD) map of the nine GAD1 SNPs in the SZ cases and controls of our discovery sample. The LD of pairwise SNPs was calculated using r^2^ algorithm implemented in the Haploview program.

In the discovery sample (528 SZ cases and 528 controls), the genotypic distributions of ten SNPs were examined. Nine SNPs were in Hardy-Weinberg equilibrium (HWE *P* > 0.05) in controls, and one SNP rs3791860 with HWE *P* < 1.00 × 10^–5^ in both cases and controls was dropped out from further analyses (Supplementary Table 1). LD structures of these SNPs in the SZ cases and controls of our discovery sample were shown in Figure 1. Logistic regression analysis found that two SNPs (rs2241165 and rs2241164) showed nominal associations with SZ (rs2241165, *P* = 0.0181, OR = 1.261; rs2241164, *P* = 0.0225, OR = 1.219; Table 1), but neither of them was significant after multiple correction (which is *P* = 2.75 × 10^–4^).

**TABLE 1:** Association results of the GAD1 SNPs in our discovery and replication samples.

CHR	POS	SNP	A1/A2	Discovery sample			Replication sample			Overall meta-analysis			
				**OR**	**SE**	***P*-value**	**OR**	**SE**	***P*-value**	**OR**	**SE**	***P*-value**	***I*^2^**
2	171672623	rs6755370	T/C	1.015	0.099	0.882	0.927	0.058	0.189	0.948	0.050	0.291	0
2	171678379	rs2241165	C/T	1.261	0.098	0.0181	1.024	0.059	0.689	1.082	0.050	0.119	64.63
2	171678625	rs11542313	C/T	0.935	0.089	0.450	0.997	0.052	0.960	0.981	0.045	0.668	48.4
2	171682740	rs3828275	T/C	0.934	0.111	0.540	1.083	0.066	0.229	1.042	0.057	0.468	15.85
2	171683758	rs3791862	A/C	0.967	0.098	0.731	0.936	0.058	0.257	0.944	0.050	0.250	0
2	171686559	rs2241164	T/C	1.219	0.087	0.0225	1.110	0.052	0.0462	1.138	0.045	0.00389	0
2	171695070	rs3791853	G/A	0.955	0.104	0.657	0.910	0.060	0.118	0.921	0.052	0.115	0
2	171709521	rs769393	A/G	0.914	0.099	0.362	1.045	0.061	0.474	1.007	0.052	0.896	0
2	171716803	rs769395	G/A	0.845	0.089	0.0565	0.969	0.054	0.562	0.933	0.046	0.136	0

*CHR, chromosome; POS, position; SNP, single nucleotide polymorphism; A1, effect allele; A2, non-effect allele; OR, odds ratio.*

**FIGURE 2:**
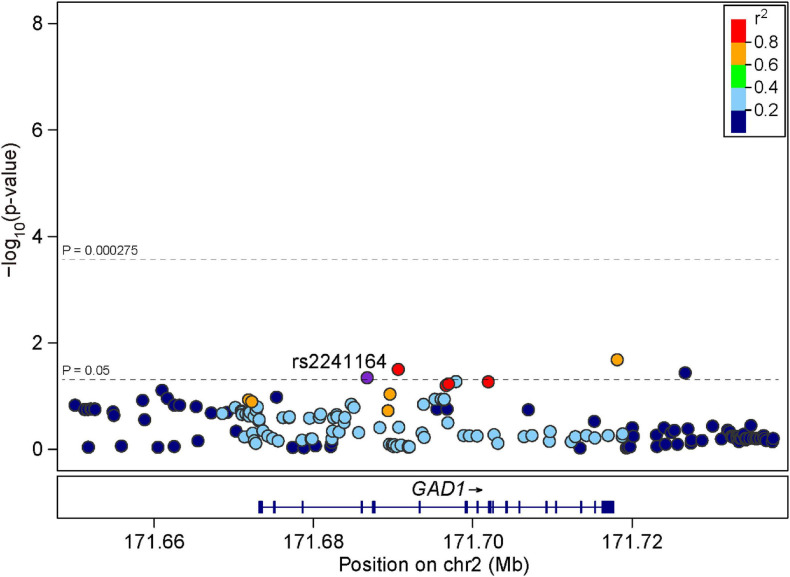
Regional association plots for GAD1 locus in the replication sample. LD information was derived from East Asian individuals in 1000 Genomes Project Phase 3. The LD is defined based on the SNP rs2241164.

We then examined the same nine SNPs within GAD1 in the independent replication sample of 1,256 early onset SZ cases and 2,661 controls. We found that rs2241164 was also nominally associated with SZ in the replication sample (*P* = 0.0462, OR = 1.110), but it did not survive multiple corrections (Table 1). We also retrieved 173 additional SNPs spanning GAD1 to examine their associations with SZ in the replication sample. Although some SNPs exhibited nominal associations with SZ (*P* < 0.05), none of the SNP-associations could survive multiple correction (Figure 2).

To maximize the statistical power of our present sample, we also performed a meta-analysis of the nine SNPs in the total sample combining both discovery and replication samples. While the association between rs2241164 and SZ became stronger after the meta-analysis (*P* = 0.00398, OR = 1.138, Table 1), it could not survive multiple correction. The other nine SNPs showed no evidence of associations with SZ in the pooled samples (all *P* > 0.05, Table 1), suggesting that they were unlikely risk factors for SZ at least in Han Chinese populations.

## Discussion

A classical etiological hypothesis of SZ is the glutamatergic neuronal dysfunction (Moghaddam and Javitt, 2012), and correlation between abnormal neural transmission in GABAergic circuits and SZ has been confirmed by accumulating studies (Devor et al., 2017). In vertebrates, the GABA synthesis enzyme GAD exists in two isoforms (GAD67 and GAD65) that are encoded by two distinct genes GAD1 and GAD2, respectively (Erlander et al., 1991). GAD67 was found to be the main rate-limiting enzyme of GABA, and down-regulation of the GAD1 gene was hence hypothesized to cause decrease of this inhibitory neurotransmitter and alterations of GABAergic circuits in the brain (Akbarian and Huang, 2006; Brown et al., 2015). Indeed, altered expression of GAD1 has been frequently reported in the SZ brains (Bharadwaj et al., 2013; Mitchell et al., 2015).

We thus sought to analyze the correlation between GAD1 SNPs and SZ in two independent Han Chinese SZ case-control samples. We have observed that certain SNPs spanning GAD1 showed nominal associations with SZ, but they were not statistically significant after multiple corrections either in each individual sample or in the pooled samples. Therefore, GAD1 is unlikely a robust risk gene for SZ in Han Chinese populations. Similarly, non-significant associations between GAD1 SNPs and SZ were reported in a previous Japanese case-control sample (Ikeda et al., 2007). To further verify these results in a larger sample, we also examined SNPs spanning GAD1 in a large GWAS of SZ in East Asians (22,778 cases and 35,362 controls primarily recruited from Han Chinese populations), but no significant SNPs surpassing the *P*-value threshold of 2.75 × 10^–4^ (Supplementary Figure 1) were identified (Lam et al., 2019).

We then examined the associations of GAD1 SNPs and SZ susceptibility in European populations. Although several candidate gene studies have reported positive associations between GAD1 and SZ (Addington et al., 2005; Straub et al., 2007), this genetic locus and its SNPs were not highlighted either in Europeans (40,675 cases and 64,643 controls) or in diverse ancestry populations (69,369 cases and 236,642 controls) in two recent large SZ GWAS (Supplementary Figures 2, 3; Pardinas et al., 2018; Ripke et al., 2020). Since these GWAS have undoubtedly sufficient statistical power in the identification of significant risk loci for SZ, we may conclude that the contribution of GAD1 in the genetic susceptibility of SZ in general populations is minimal.

While our analysis did not reveal robust statistical associations between GAD1 SNPs and SZ, it raised a possibility that a gene with putative roles in pathophysiological processes linked with SZ may not always be a strong genetic risk factor (Sullivan, 2007, 2017). In fact, GAD1 is not the only gene supporting this contention. Majority of the genes, which encode proteins with pivotal roles in physiological pathways related to SZ, were extensively analyzed during the earlier era of candidate gene studies in SZ genetics, yet these genes did not show significant associations in later SZ GWAS (Farrell et al., 2015; Johnson et al., 2017). Nevertheless, this inconsistency does not deny potential impact of these genes on SZ. Modulation of their mRNA levels or epigenetic signatures, by undetermined genetic or environmental factors, may still confer risk of SZ. For example, Tao et al. have reported significant differences of GAD1 mRNA expression and epigenetic modifications between SZ cases and controls (Tao et al., 2018).

Notably, inconsistencies between genetic studies in smaller samples and in large GWAS are often observed, i.e., SNPs of certain genes (such GAD1) may be significant in smaller samples, but not significant in large GWAS cohorts. This may be a result of the etiological and phenotypic heterogeneity of the illness. Specifically, such SNPs/genes may participate in the pathogenesis of certain subtype(s) of SZ, whereas these subjects may take up only a limited proportion in large GWAS studies that usually do not stratify samples based on phenotypic characteristics. In the present study, the discovery sample comprised of paranoid SZ cases, and the replication sample included early onset SZ patients. Therefore, the non-significance of the results may actually reflect involvement of GAD1 in certain subtype(s) of SZ remain to be characterized. Future analyses exploring the correlation between GAD1 and various subtypes of SZ in larger Han Chinese samples with detailed phenotyping are thus warranted.

## Conclusion

In summary, our study revealed a weak association between GAD1 SNPs and risk of SZ in Han Chinese populations. Further analyses of SNPs in this gene in different certain subtypes of SZ with detailed phenotyping may provide useful insights about the role of this gene in SZ pathogenesis.

## Data Availability Statement

The datasets generated for this study are available on https://www.oebiotech.com/index.php?c=show&id=352.

## Ethics Statement

The studies involving human participants were reviewed and approved by the Ethics Committee of the Second Affiliated Hospital of Xinxiang Medical University. The patients/participants provided their written informed consent to participate in this study.

## Author Contributions

WL and LL designed the study protocol. LZ, ZL, QL, MiS, and YY managed the literature searches and analyses. YY, FS, MeS, XS, and YZ conducted sample selection and data management. YZ, MD, YL, and WL undertook the statistical analysis. ML and JL provided advice and discussion during the design and revision of the present study. LZ, WL, ML, and YY wrote the first draft of the manuscript. All authors contributed to and have approved the final manuscript.

## Conflict of Interest

The authors declare that the research was conducted in the absence of any commercial or financial relationships that could be construed as a potential conflict of interest.

## Footnotes

1 http://locuszoom.sph.umich.edu/locuszoom/
