# Supplementary material for: Weak Association Between the Glutamate Decarboxylase 1 Gene (GAD1) and Schizophrenia in Han Chinese Population
Source: Front Neurosci. 2021 Jun 21;15:677153. doi: 10.3389/fnins.2021.677153 (PMC8255988; doi:10.3389/fnins.2021.677153)
Supplement: Supplementary file 1 [file Table_1.DOCX]

**Table S1. Genotype and allele frequencies of** [**t**](app:ds:thirty)**en SNPs** **within the GAD1 gene in SZ cases and controls of discovery sample.**

| **CHR** | **POS** | **SNP** | **A1/A2** | **Diagnosis** | **N** | **HWE** | **Genotype counts** | | | **Genotypic P-value** | **Allele counts** | | **Allelic**  **P-value** |
| --- | --- | --- | --- | --- | --- | --- | --- | --- | --- | --- | --- | --- | --- |
|  |  |  |  |  |  |  | **A1/A1** | **A1/A2** | **A2/A2** |  | **A1** | **A2** |  |
| 2 | 171672623 | rs6755370 | T/C | Case | 527 | 0.140 | 42 | 189 | 296 | 0.198 | 273 | 781 | 0.881 |
|  |  |  |  | Control | 527 | 0.360 | 30 | 210 | 287 |  | 270 | 784 |  |
| 2 | 171678379 | rs2241165 | C/T | Case | 527 | 1.000 | 48 | 222 | 257 | 0.0554 | 318 | 736 | 0.0184 |
|  |  |  |  | Control | 528 | 0.647 | 32 | 206 | 290 |  | 270 | 786 |  |
| 2 | 171678625 | rs11542313 | C/T | Case | 527 | 0.930 | 104 | 259 | 164 | 0.521 | 467 | 587 | 0.455 |
|  |  |  |  | Control | 528 | 0.293 | 105 | 275 | 148 |  | 485 | 571 |  |
| 2 | 171682740 | rs3828275 | T/C | Case | 528 | 0.665 | 16 | 163 | 349 | 0.694 | 195 | 861 | 0.542 |
|  |  |  |  | Control | 528 | 0.782 | 21 | 164 | 343 |  | 206 | 850 |  |
| 2 | 171683758 | rs3791862 | A/C | Case | 528 | 0.364 | 39 | 194 | 295 | 0.781 | 272 | 784 | 0.729 |
|  |  |  |  | Control | 528 | 1.000 | 37 | 205 | 286 |  | 279 | 777 |  |
| 2 | 171686559 | rs2241164 | T/C | Case | 528 | 0.0238 | 140 | 238 | 150 | 0.00319 | 518 | 538 | 0.0208 |
|  |  |  |  | Control | 528 | 0.217 | 95 | 275 | 158 |  | 465 | 591 |  |
| 2 | 171689421 | rs3791860 | C/G | Case | 528 | 1.39×10^−6^ | 0 | 159 | 369 | NA | 159 | 897 | NA |
|  |  |  |  | Control | 527 | 8.69×10^−6^ | 1 | 161 | 365 |  | 163 | 891 |  |
| 2 | 171695070 | rs3791853 | G/A | Case | 527 | 0.607 | 26 | 174 | 327 | 0.856 | 226 | 828 | 0.652 |
|  |  |  |  | Control | 528 | 0.317 | 30 | 175 | 323 |  | 235 | 821 |  |
| 2 | 171709521 | rs769393 | A/G | Case | 527 | 0.576 | 40 | 200 | 287 | 0.152 | 280 | 774 | 0.368 |
|  |  |  |  | Control | 528 | 0.0862 | 34 | 231 | 263 |  | 299 | 757 |  |
| 2 | 171716803 | rs769395 | G/A | Case | 526 | 0.0552 | 75 | 219 | 232 | 0.0604 | 369 | 683 | 0.0511 |
|  |  |  |  | Control | 527 | 1.000 | 81 | 251 | 195 |  | 413 | 641 |  |

**Abbreviation:** CHR, chromosome; POS, position; SNP, single nucleotide polymorphism; A1, effect allele; A2, non-effect allele.

**Figure S1. Regional association plots for GAD1 locus in the SZ GWAS of East Asian individuals (22,778 cases and 35,362 controls) (**[**Lam et al., 2019**](#_ENREF_1)**).** LD information was derived from East Asian individuals in 1000 Genomes Project Phase 3. The LD is defined based on the SNP rs2241164.


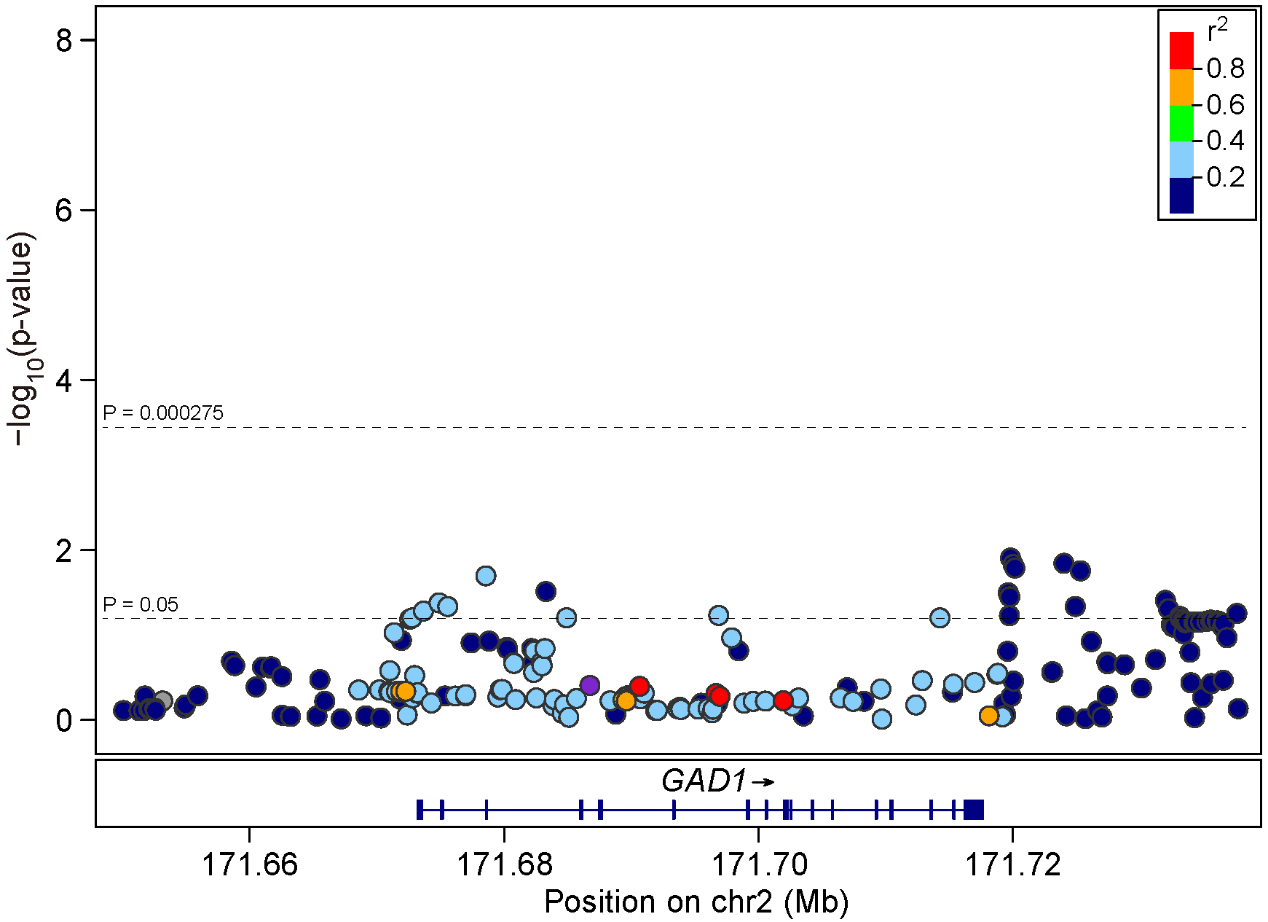


**Figure S2. Regional association plots for GAD1 locus in the SZ GWAS of European individuals (40,675 cases and 64,643 controls) (**[**Pardinas et al., 2018**](#_ENREF_2)**).** LD information was derived from European individuals in 1000 Genomes Project Phase 3. The LD is defined based on the SNP rs2241164.


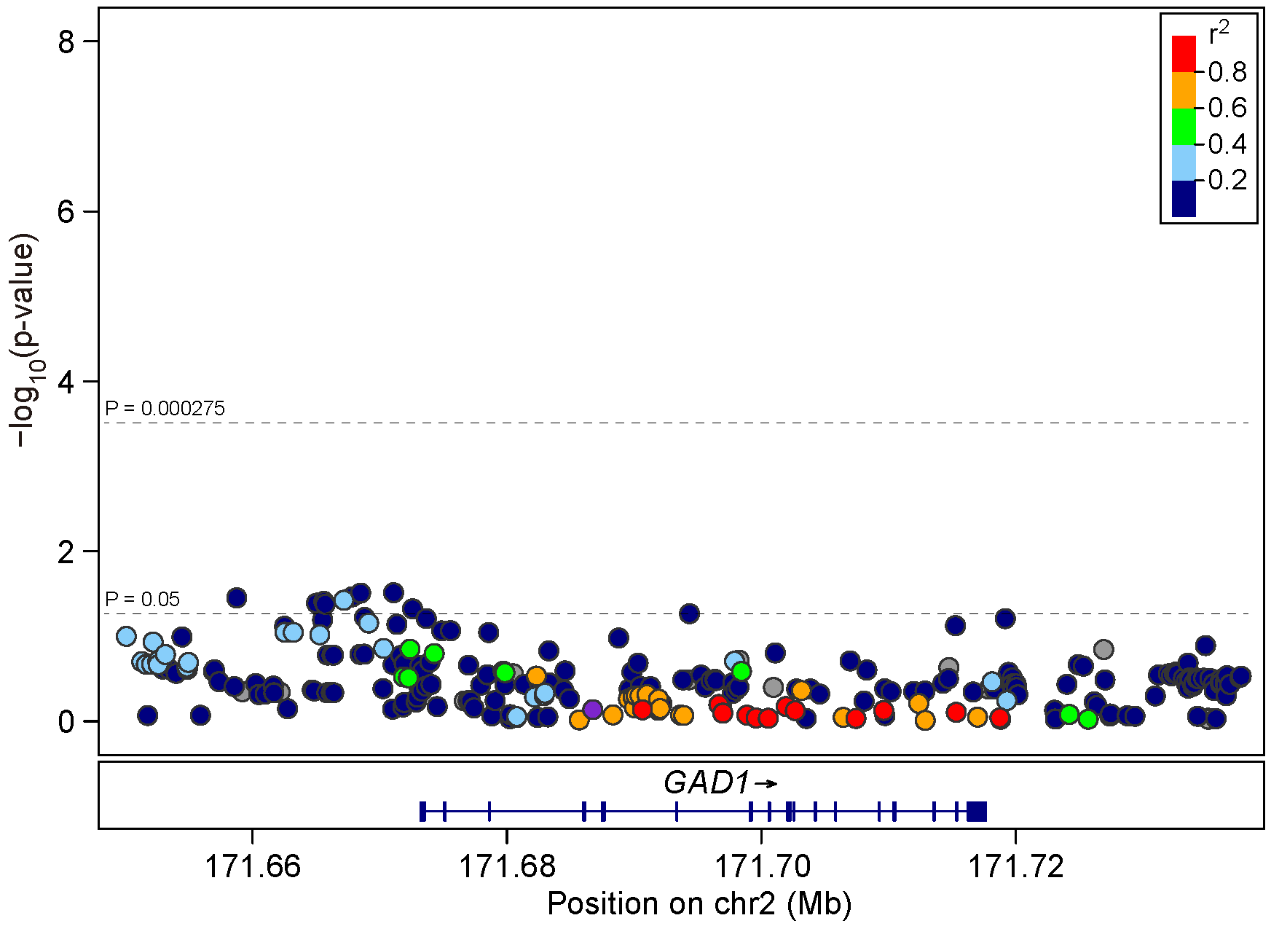


**Figure S3. Regional association plots for GAD1 locus in the SZ GWAS of diverse population individuals (69,369 cases and 236,642 controls) (**[**Ripke et al., 2020**](#_ENREF_3)**).** LD information was derived from European individuals in 1000 Genomes Project Phase 3. The LD is defined based on the SNP rs2241164.


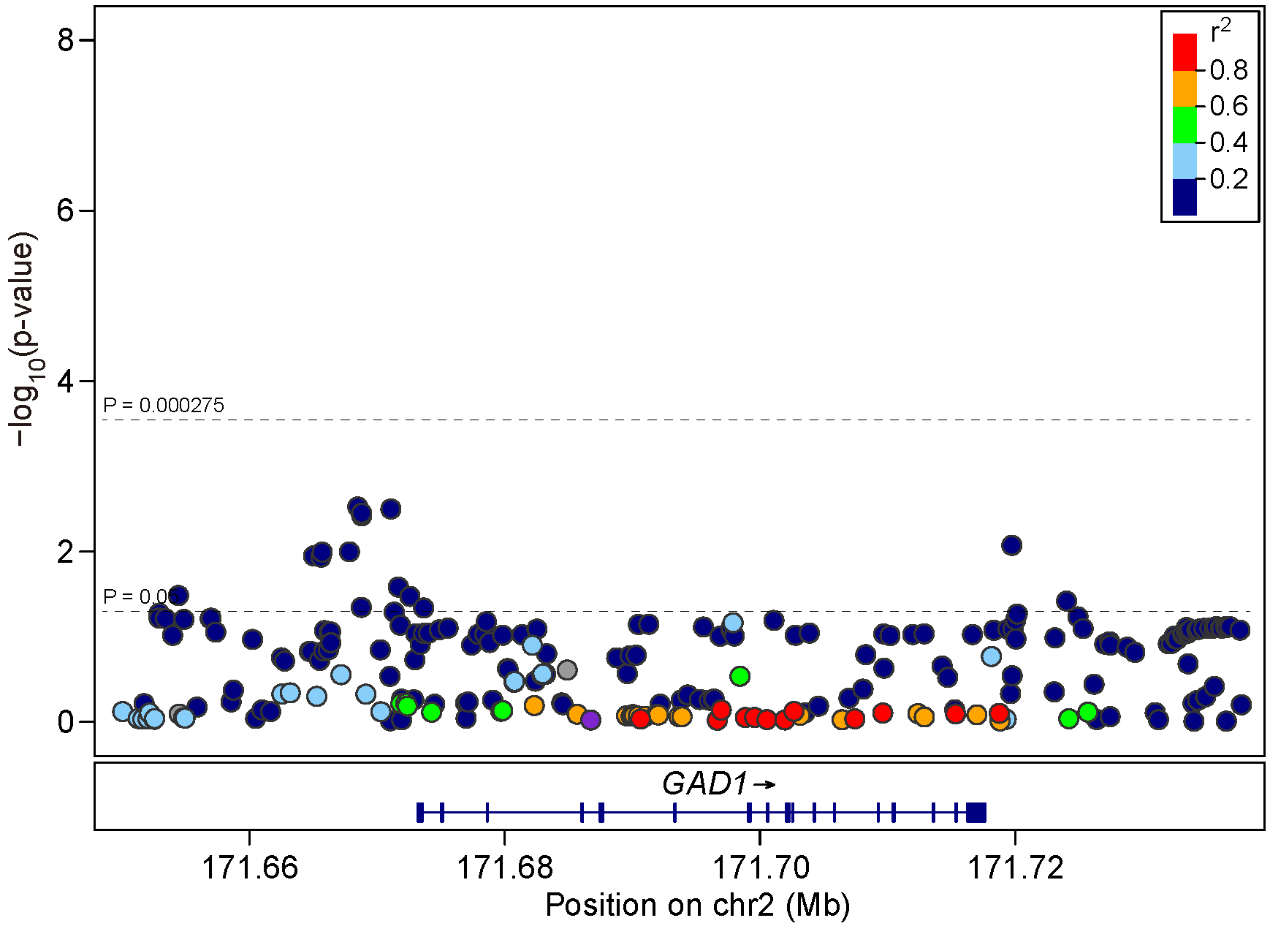


**References**

Lam, M., Chen, C.Y., Li, Z., Martin, A.R., Bryois, J., Ma, X., et al. (2019). Comparative genetic architectures of schizophrenia in East Asian and European populations. *Nat Genet* 51**,** 1670-8.

Pardinas, A.F., Holmans, P., Pocklington, A.J., Escott-Price, V., Ripke, S., Carrera, N., et al. (2018). Common schizophrenia alleles are enriched in mutation-intolerant genes and in regions under strong background selection. *Nat Genet* 50**,** 381-9.

Ripke, S., Walters, J.T., O'donovan, M.C., and Schizophrenia Working Group of the Psychiatric Genomics Consortium (2020). Mapping genomic loci prioritises genes and implicates synaptic biology in schizophrenia. *MedRxiv*.
